# A first principle investigation to explore the effect of Zr-site Ti doping on structural, electronic, optical, and mechanical properties of BaZrO_3_

**DOI:** 10.1038/s41598-025-11576-9

**Published:** 2025-07-21

**Authors:** Apon Kumar Datta, M. Khalid Hossain, M. S. Revathy, M. Sudhakara Reddy, Abhayveer Singh, S. Radhika, Satish Choudhury, Ankur Singh Bisht, Abdullah M. S. Alhuthali, Magda H. Abdellattif, R. Balachandran, Rajesh Haldhar

**Affiliations:** 1 Department of Electrical and Electronic Engineering, Mymensingh Engineering College, Mymensingh, 2200 Bangladesh; 2 Institute of Electronics, Atomic Energy Research Establishment, Bangladesh Atomic Energy Commission, Dhaka, 1349 Bangladesh; 3 Department of Advanced Energy Engineering Science, Interdisciplinary Graduate School of Engineering Sciences, Kyushu University, Fukuoka, 816-8580 Japan; 4 Department of Physics, School of Advanced Sciences, Kalasalingam Academy of Research and Education, Krishnankoil, Virudhunagar, Tamil Nadu 626126 India; 5 Department of Physics & Electronics, School of Sciences, JAIN (Deemed to Be University), Bangalore, Karnataka India; 6 Centre for Research Impact & Outcome, Chitkara University Institute of Engineering and Technology, Chitkara University, Rajpura, 140401 Punjab India; 7 Department of Electrical and Electronics Engineering, Sathyabama Institute of Science and Technology, Chennai, Tamil Nadu India; 8 Department of Electrical & Electronics Engineering, Siksha ‘O’ Anusandhan (Deemed to Be University), Bhubaneswar, Odisha 751030 India; 9 Department of Computer Science and Engineering, Graphic Era Hill University, Bhimtal, Uttarakhand 248002 India; 10 Graphic Era Deemed to Be University, Dehradun, Uttarakhand 248002 India; 11 Department of Physics, College of Sciences, Taif University, P.O. Box 11099, 21944 Taif, Saudi Arabia; 12 Department of Chemistry, College of Science, University College of Taraba, Taif University, P.O. Box 11099, Taraba, Saudi Arabia; 13 Department of ECE, College of Electrical Engineering and Computing, Adama Science and Technology University, P.O.Box No. 1888, Adama, Ethiopia; 14 School of Chemical Engineering, Yeungnam University, Gyeongsan, 38541 Republic of Korea

**Keywords:** BaZrO_3_ perovskite, Density functional theory (DFT), Transitional metal, Doping, Bandgap tuning, Materials science, Optics and photonics, Physics

## Abstract

Doped BaZrO_3_ is well recognized as a promising material for proton conduction, particularly in solid oxide fuel cells (SOFCs) and various electrochemical applications. While this material has been thoroughly examined for proton conduction, it has not been as extensively studied for other potential applications, such as photocatalytic water splitting and solar cell devices. This investigation delves into the comprehensive assessment of structural, electronic, optical, mechanical, and thermodynamic properties in Ti-doped BaZrO_3_ (BaZr_1-x_Ti_x_O_3_ where x = 0,0.25, 0.5, 0.75) through the application of Density Functional Theory (DFT) employing the Generalized Gradient Approximation (GGA) and Perdew-Burke-Ernzerhof (PBE) exchange–correlation function. After doping, all of the doped compounds undergo a phase transition from cubic to tetragonal once Ti is added to BaZrO_3_. Analysis of the computed structural properties reveals a slight reduction in lattice parameters accompanied by a decrease in cell volume. The doping of Ti led to a reduction in the electronic bandgap energy of BaZrO_3_. Specifically, the bandgap decreased from an initial value of 3.118 eV at x = 0, which was an indirect bandgap, to a lowest value of 1.8 eV at x = 0.5, also identified as an indirect bandgap. This bandgap reduction leads to significant changes in optical properties, enabling absorption at lower photon energies compared to pure BaZrO_3_, which is beneficial for photocatalytic water splitting and solar cell applications. Mechanical properties confirmed the stability of the investigated composition through the Born stability criteria. Furthermore, thermodynamic properties across different doping concentrations revealed the highest Debye temperature at x = 0.75, indicating a higher melting point and enhanced thermal stability.

## Introduction

Transitional metal (TM) perovskite oxides represent a critically significant class of materials that garnered substantial attention both theoretically and experimentally across numerous fields of material science, owing to their profound technological applications. Perovskite oxides with the general formula ABO_3_, where the B-site is occupied by a transition metal cation and the A-site by a rare earth cation, exhibit exceptional versatility with applications spanning photocatalysis, multiferroic devices, superconductors, thermoelectric converters, and advanced energy storage systems^1–6^. As a result, across a wide range of technologies, including microwave devices, optoelectronic modulators, solar cells, fuel cells, infrared sensors, infrared detectors, and other electromechanical systems, the ABO_3_ perovskite has proven to be adaptable^7–9^. Additionally, TM perovskite oxides have been examined and found to have several advantages, including a large bandgap and good carrier mobility. These qualities make them attractive candidates for a range of uses, including high-frequency, high-power, and transparent conductor deployment^10^. By carefully adjusting their transport, magnetic, and optical characteristics by doping, strain engineering, or external fields, specific functions for particular applications are made possible. This tunability makes them suitable for integration into devices such as metal–semiconductor field-effect transistors, solar cell absorber layers, and photocatalytic systems for water-splitting applications^11–13^. Solid oxide fuel cells (SOFC) are a highly regarded energy conversion technology that has attracted a lot of interest in terms of performance enhancement and commercialization^14^. Operating SOFC at high temperatures (> 1000 °C) often leads to challenges such as high production costs, material degradation, reduced performance, limited stability, and shortened device lifespan. Among high-temperature proton-conducting materials, TM oxide perovskite BaZrO_3_ (BZO) has garnered attention, though its practical use is hindered by low conductivity due to grain boundary blocking and poor sinterability^15–30^. However, doping BZO with lower-valence cations such as yttrium (Y) has been shown to mitigate these issues which improved conductivity and stability at reduced operating temperatures^31–33^. Moreover, high proton conductivity, coupled with commendable chemical and mechanical stability, positions acceptor-doped perovskite oxides as highly promising contenders for serving as electrolytes in low-temperature solid oxide fuel cells (SOFCs)^34–37^.

In addition to their use in SOFCs, wide bandgap transition metal oxide perovskites are also emerging as promising absorber materials for solar cell devices and photocatalysts for water-splitting applications^38,39^. In TM oxide compounds, the valence band is typically formed by the 2p orbitals of oxygen atoms, while the conduction band mainly arises from the d orbitals of the TM-site cation. The degree of interaction between the TM-site atom and oxygen is influenced by the electronegativity of the TM-site element. As a result, the nature of the B-site component plays a crucial role in determining the photocatalytic performance of perovskite materials^40–42^. For example, previous studies have shown that TiO_2_, with its wide band gap of 3.2 eV, is limited to UV light absorption. However, doping with transition metals such as Cr or Ni can effectively narrow the band gap, enabling visible light sensitivity and improving carrier separation^3^. Another study on SrTiO_3_ TM perovskite oxide showed that incorporating Fe cations effectively tunes its band gap, making it suitable as an absorber layer for photovoltaic applications. Unlike pure STO, which absorbs only in the UV region (λ < 400 nm), Fe-doped STO displays a broad, intense absorption from 0.5 to 6 eV, enabling strong visible light activity. Moreover, according to the estimated band gap of the Fe-doped STO sample (1.43 eV), they suggested Nanocrystalline SrTi_0.9_Fe_0.1_O_2.968_ (STFO) for photocatalytic application^1^. Hydrogen production relies on photogenerated electrons and holes in a material to drive water oxidation and reduction^4^. The efficiency of a photocatalyst depends on the positions of its conduction and valence band edges relative to the redox potentials of water. Ideally, materials with a band gap above 1.23 eV can drive half-reactions required for water splitting^43^. Since just 4% of solar energy is in the ultraviolet light area, a photocatalyst must absorb substantially in the visible range of the spectrum in order to be energy-efficient. However, wide band gap materials cannot utilize visible light effectively due to a higher optical bandgap. Therefore, tuning the band edges is a possible way to make a photocatalyst suitable for water splitting under visible light. Cr-doped SrTi_1-x_Cr_x_O_3_ (x = 0.00, 0.02, 0.05, 0.10) powders were synthesized in a previous study using the solvothermal method. The introduction of chromium into the TM oxide SrTiO_3_ lattice results reduction in the band gap, which falls within the visible light range. This new bandgap played a significant role in enhancing the absorption of visible light absorption which is attributed to electronic transitions from Cr 3 d states to hybridized Cr 3 d + Ti 3 d orbitals. Such improved absorption characteristics are particularly beneficial for the application of SrTiO_3_ as a photocatalyst in water-splitting processes^44^. Previous studies have also employed both experimental and computational approaches to investigate metal and nonmetal mono-doping, as well as co-doping, into potential transition metal oxide perovskites, including BaTiO_3_, CaTiO_3_, CaZrO_3_, and CaZrO_3_^45–55^. These investigations have demonstrated that such modifications can tune the bandgap properties, making the wide bandgap TM oxide perovskites promising for photocatalytic water splitting applications by altering the conduction and valence band edges.

TM oxide zirconium-based perovskite oxides, especially BaZrO_3_, have been extensively studied in recent years due to their potential for thermoelectric and optoelectronic applications^32,33^. BaZrO_3_ is well recognized for its role in proton-conducting solid oxide fuel cells^56^. Because of their high melting points and excellent mechanical and thermal durability across a wide temperature range, zirconates have long attracted interest for use in thermal barrier coatings. Additionally, BaZrO_3_ doped with rare-earth elements such as Y, Yb, In, Sc, Gd, Nd, and Sm has been widely investigated as a proton-conducting electrolyte, where the optimization of dopant type and concentration significantly enhances proton conductivity^28,57,58^. Although doped BaZrO_3_ has been studied for optoelectronic uses according to previous literature, there is still a lack of sufficient deep understanding of how doping influences its electrical structure, particularly regarding bandgap narrowing and the possibility of improving its optoelectronic characteristics. More specifically, its viability as a photocatalyst or solar cell absorber material has yet to be thoroughly explored.

The primary objective of this investigation is to determine the relationship between the physical properties of BaZr_1-x_Ti_x_O_3_ and its suitability as an absorber layer for solar cells and photocatalytic applications. A doping strategy has been employed to tune the bandgap of BaZrO_3_, thereby modifying its optical properties to make it suitable for photovoltaic and photocatalytic applications under visible light. Transition metal titanium (Ti) is used to substitute zirconium (Zr) atoms in the BaZrO_3_ perovskite lattice, enabling orbital hybridization between Zr-4d and Ti-3d states. This interaction can effectively narrow the energy gap between the conduction band minimum (CBM) and valence band maximum (VBM). Doping concentrations of BaZr_1-x_Ti_x_O_3_ (where x = 0, 0.25, 0.5, and 0.75) were modeled through atomic substitution in a 2 × 2 × 1 supercell. All calculations were carried out using Density Functional Theory (DFT) with the GGA-PBE exchange–correlation functional^59–65^.

## Computational details

**Fig. 1:**
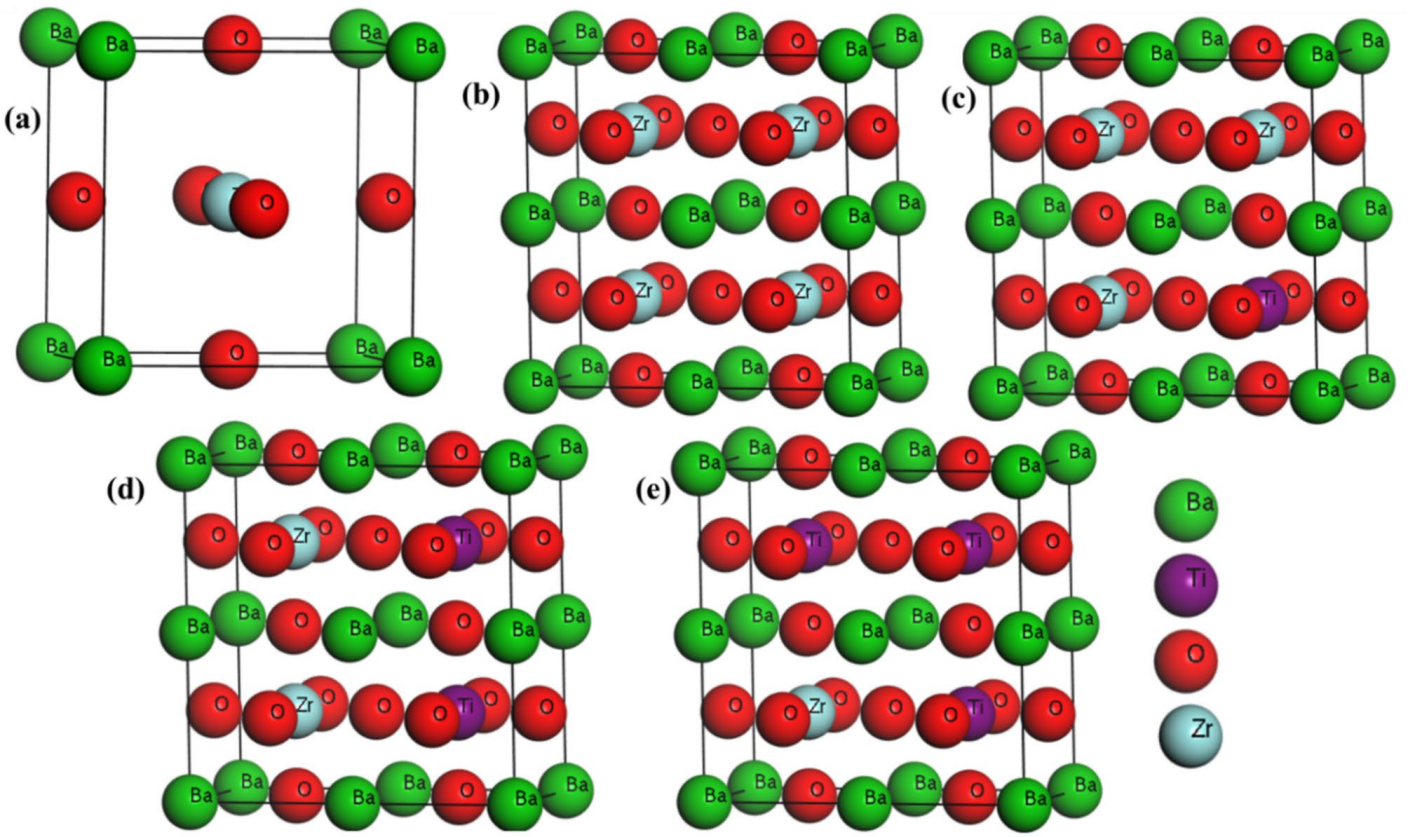
(**a**) Unit cell structure of BaZrO_3_, and different atomic arrangement in BaZ_r-x_Ti_x_O_3_ for (**b**) x = 0, (**c**) x = 0.25, (**d**) x = 0.5, and (**e**) x = 0.75.

This study of analyzing material properties has been conducted using the Cambridge Serial Total Energy Package (CASTEP), developed by Payne and others in the 1990s. This calculation is based on DFT, which is performed using Plane Wave Psudopotential basis set with the ultrasoft pseudopotential (USP)^66,67^. To explore the ground state structure, the Broyden–Fletcher–Goldfarb–Shanno (BFGS) method has been employed. Geometry optimization has been carried out in this work using the generalized gradient approximation (GGA) with the Perdew-Burke-Ernzerhof (PBE) function^68^. This study employed a cubic structure of BaZrO_3_ and a 2 × 2 × 1 supercell, as shown in Fig. 1. To introduce an impurity, Zr atoms were replaced by Ti in the supercell structure, corresponding to a doping concentration of 25%, 50%, and 75%. Consequently, the chemical formula of the BaZrO_3_ perovskite was modified to BaZ_r-x_Ti_x_O_3_ (where x = 0, 0.25, 0.5, 0.75). A cutoff of energy 300 (eV) has been used, along with a k-mesh grid of 3 × 3 × 3 for conducting calculations within the irreducible Brillouin zone. Configuration parameters included 1 × 10^–3^ Å as maximum displacement, 0.05 GPa for stress, and a tolerance for force per atom set at 5.0 × 10^−6^ eV/Å. The electronic and optical properties are computed based on the aforementioned parameters utilized in structural optimization. Thermal properties are explained through the analysis of mechanical properties.

## Result and discussion

### Structural properties

The investigation involves the cubic oxide perovskite BaZrO_3,_ which falls within the crystallographic space group of Pm3m (No. 221). In the BZO unit cell, the atoms are distributed as follows: Ba atoms situated at the corner positions with fractional coordinates of (0,0,0), B site atoms occupying the body-centered position with fractional coordinates of (0.5,0.5,0.5), and O atoms positioned at the face-centered locations with fractional coordinates of (0,0.5,0.5), (0.5,0,0.5), and (0.5,0.5,0).

Figure 1 illustrates the BaZrO_3_ unit cell, a pure 2 × 2 × 1 supercell structure of BaZrO_3_, and Ti-doped BaZrO_3_ supercell structures. After geometry optimization, the calculated lattice parameter of pure BaZrO_3_ is well-matched with previously studied theoretical and experimental works, which is illustrated in Table 1^16^. Detailed geometrically optimized structural parameters for both the pure and Ti-doped BZO are provided in Table 1**.** Due to the Ti doping in BZO has lessened the lattice parameters have lessened overall, which may be due to the smaller ionic radius of Ti (0.067 nm) than Zr (0.080 nm). Moreover, when the Ti dopant was added to the Zr site of BaZrO_3_, the change of phase was observed. After Ti doping at different levels, the cubic symmetry of BZO with a space group of Pm3m changes to Tetragonal P4/mm symmetry.

**Table 1:** Optimized lattice parameters a_0_ (Å) and cell volume V_0_ (Å^3^) of BaZ_r-x_Ti_x_O_3_.

Composition	Symmetry	Function	a_0_ (Å)	V_0_ (Å^3^)	Remarks
Pure BaZrO_3_	Pm3m	GGA	4.26	77.30	Cal^16^.
	Pm3m	–	4.19	73.61	Expt^23^.
	Pm3m	GGA	4.223	73.5	Cal^19^.
	Pm3m	–	4.191	–	Expt^25^.
	Pm3m	–	4.192	73.66	Expt^26^.
	Pm3m	LDA	4.154	71.68	Cal^30^.
	Pm3m	LDA	4.16	72.30	Cal^20^.
	Pm3m	GGA	4.25	77.27	This work
BaZr_0.75_Ti_0.25_O_3_	P4/mm	GGA	a = b = 8.42 c = 4.23	300.63	This work
BaZr_0.5_Ti_0.5_O_3_	P4/mm	GGA	a = b = 4.18 c = 8.27	290.04	This work
BaZr_0.25_Ti_0.75_O_3_	P4/mm	GGA	a = b = 8.21 c = 4.12	278.41	This work

### Electronic properties

#### Electronic band structure

**Fig. 2:**
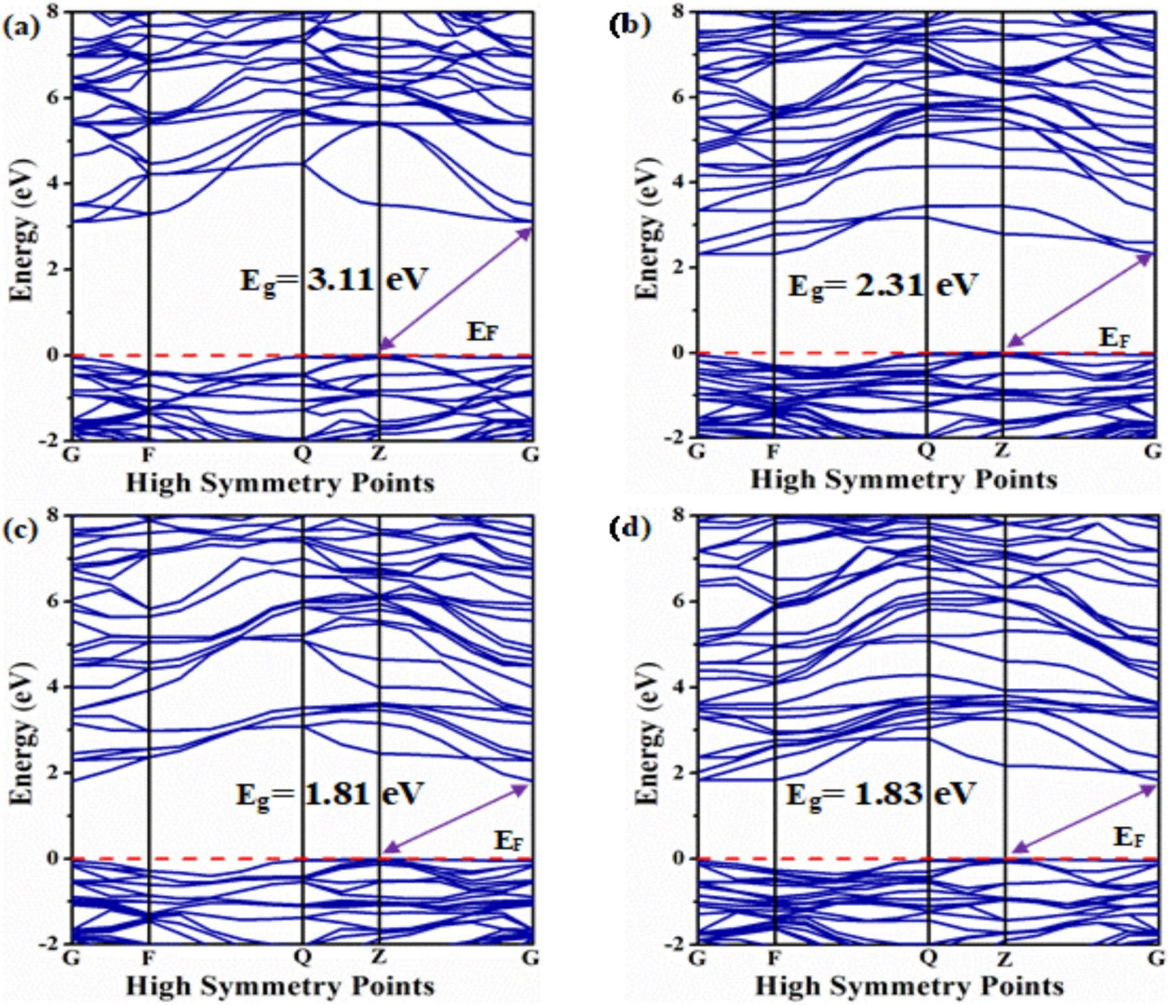
Electronic bandstructure of BaZr_1-x_Ti_x_O_3_ where (**a**) x = 0, (**b**) x = 0.25, (**c**) x = 0.5, and (**d**) x = 0.75.

The electronic response of a material is indicated by its electronic band structure and density of states (DOS) characteristics^69^. The electronic band structures along high symmetry directions have been computed to analyze the electronic properties of both undoped BZO and Ti-doped BZO as depicted in Fig. 2. Figure 2(a) illustrates the bandgap of pure BZO, while the subsequent figures (Fig. 2**(b-d)**) depict the bandgaps of BZO doped with increasing percentages of Ti, arranged in ascending order. The valence band maxima (VBM) are found at the R symmetry point, while the conduction band minima (CBM) are found at the G-symmetry point in all doping concentrations (x = 0.25, 0.5, 0.75). As the symmetry points are different in the electronic structure of the material, hence is called an indirect band gap. The Fermi energy level (E_F_) is denoted by the red dotted line. The position of the VBM remains largely consistent across all doping concentrations, with noticeable variances only apparent in the CBM position. In pure BZO, the difference between these two positions is represented by the measured band gap of 3.119 eV. The determined band gap energy of pure BZO is closely aligned with the previously postulated bandgap energy (Table 2), confirming the accuracy and reliability of this theoretical approach for band gap calculations^17^.

**Table 2:** Comparison of calculated bandgap values with previously reported studies.

Composition	Function	Bandgap	Nature	Remarks
Pure BaZrO_3_	GGA	3.119	Indirect	Cal^17^.
	GGA	3.164	Indirect	Cal^19^.
	FP-LAPW	3.23	Indirect	Cal^20^.
	GGA	3.11	Indirect	This work
BaZr_0.75_Ti_0.25_O_3_	GGA	2.31	Indirect	This work
BaZr_0.5_Ti_0.5_O_3_	GGA	1.81	Indirect	This work
BaZr_0.25_Ti_0.75_O_3_	GGA	1.83	Indirect	This work

After introducing Ti (x = 0.25) into the Zr site of BZO, the band gap experiences a notable reduction from 3.117 eV to 2.331 eV. This reduction occurs due to the shifting of the conduction band (CB) of the pure BZO compounds towards lower energy levels (redshift). The calculated band structure indicates a discernible alteration in the bandgap pattern following a 25% Ti doping into the Zr site. As the doping percentage increases (x = 0.5), a tendency of reduction in the bandgap is still observed. At a doping level of 50%, the band gap decreases to 1.81 eV. Moreover, at a higher doping concentration of 75% Ti, the band gap experiences a slight increase, reaching 1.831 eV. In all cases, the VBM is situated within Z symmetry, while the CBM aligns with G symmetry. When the valence band (VB) crosses above the Fermi energy level, figuring out the band gap value on the band structure diagram gets somewhat complex. This is a result of semiconductors having degenerate doping, which produces an excess of electron carriers over the density of states (DOS) at the conduction band edge. When degenerately doped, the Fermi level is located in the CB, which makes it difficult to determine the exact band gap value. This phenomenon is commonly referred to as the Moss–Burstein effect^70,71^. However, this effect has not been seen in this investigation. In indirect bandgap materials, direct electron transition from valence band to conduction band is not possible due to the momentum mismatch between the valence band maximum (VBM) and the conduction band minimum (CBM). Phonon-assisted interband transitions play a crucial role in overcoming this mismatch. By absorbing or emitting phonons, which vary in momentum, these transitions facilitate electron excitation across the bandgap. Interband transitions are essential for the operation of optoelectronic devices, as they involve the excitation of charge carriers from the valence band to the conduction band, which is also a key process in optoelectronic devices such as solar cells, laser diodes, and light-emitting diodes (LEDs)^72,73^.

#### Total and partial density of states

**Fig. 3:**
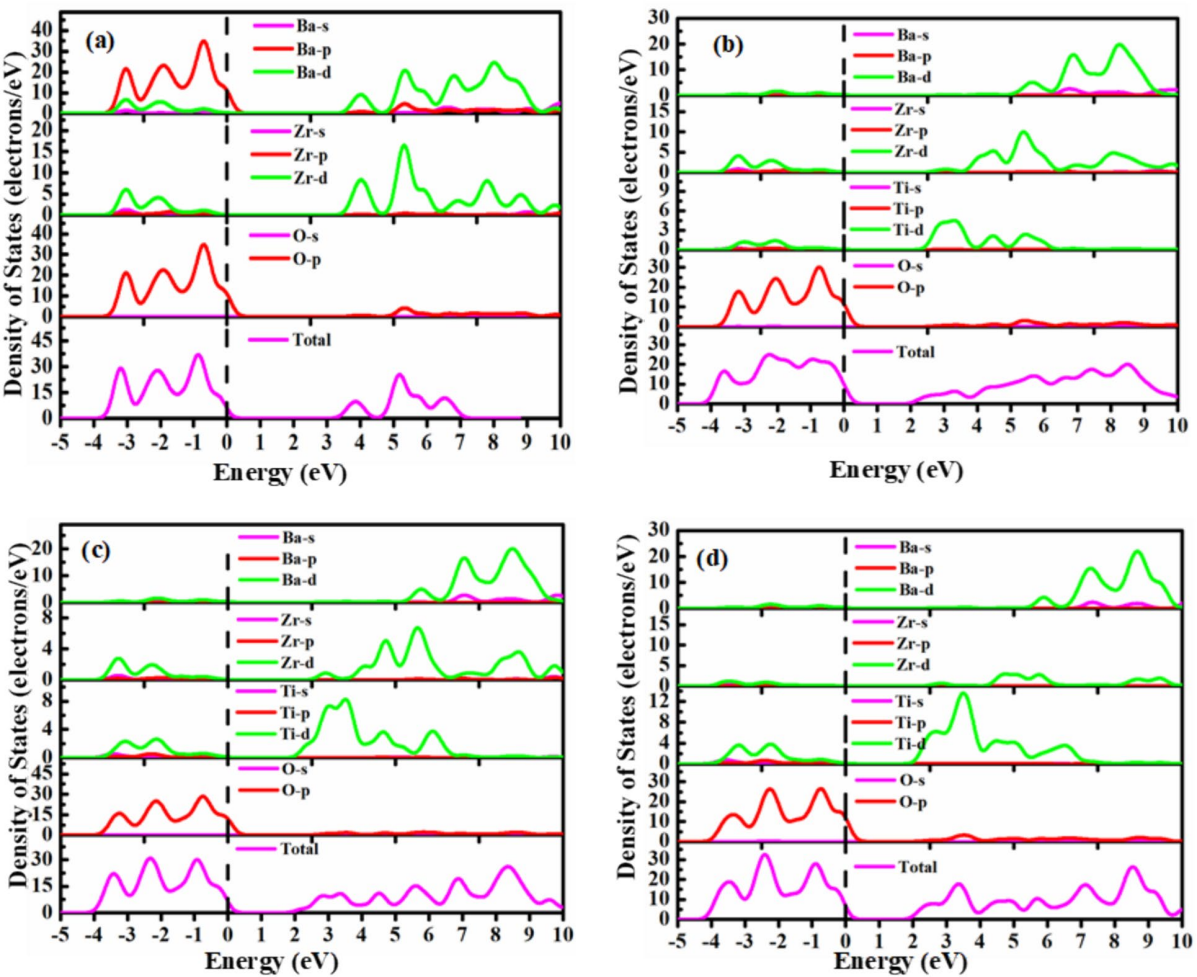
TDOS and PDOS of BaZr_1-x_Ti_x_O_3_ where (**a**) x = 0, (**b**) x = 0.25, (**c**) x = 0.5, and (**d**) x = 0.75.

The electronic density of states refers to the number of electronic energy levels available per unit of energy at each energy level, accommodating electrons within a material. Figure 3 illustrates the TDOS and PDOS for pristine BZO and Ti-doped BZO. To explore the nature of chemical bonds in both pure and doped forms, as well as the orbital contribution to the TDOS, it is needed to calculate the PDOS. The energy level corresponding to the Fermi level is indicated by the vertical dotted line positioned at 0 eV. However, the VB is specified in the lower portion of the fermi level, while the CB is shown in the upper part. Figure 3(a) shows the Density of States (DOS) of pure BZO in both its total and partial forms, which indicates that the predominant contribution to the top of the VB comes from a combination of O-2p state and Ba-p states. Meanwhile, the CBM comes from the 4d-state of Zr in pure BaZrO_3_. When Ti is doped into BZO at 25%, the O-2p orbital becomes the primary contributor of the VB in doped BZO, with a minor influence from the Zr-4d and Ti-3d states. That means the Ti-3d state is leading to form CBM in doped BZO, with minor involvement of the Zr-4d state depicted in Fig. 3** (b)**. Figure 3(c) and (d) depict the TDOS and PDOS of Ti-doped BZO at higher doping concentrations (x = 0.5, 0.75). In both doping concentrations, the 2p-states of O still play a dominant role in forming the VB, while the CB primarily originates from the Ti-3d and Zr-4d states. However, as the doping concentration increases, the potential presence of Ti-3d states has been observed, which could contribute to a reduction in the band gap from 3.11 eV to 1.81 eV.

#### Charge density

**Fig. 4:**
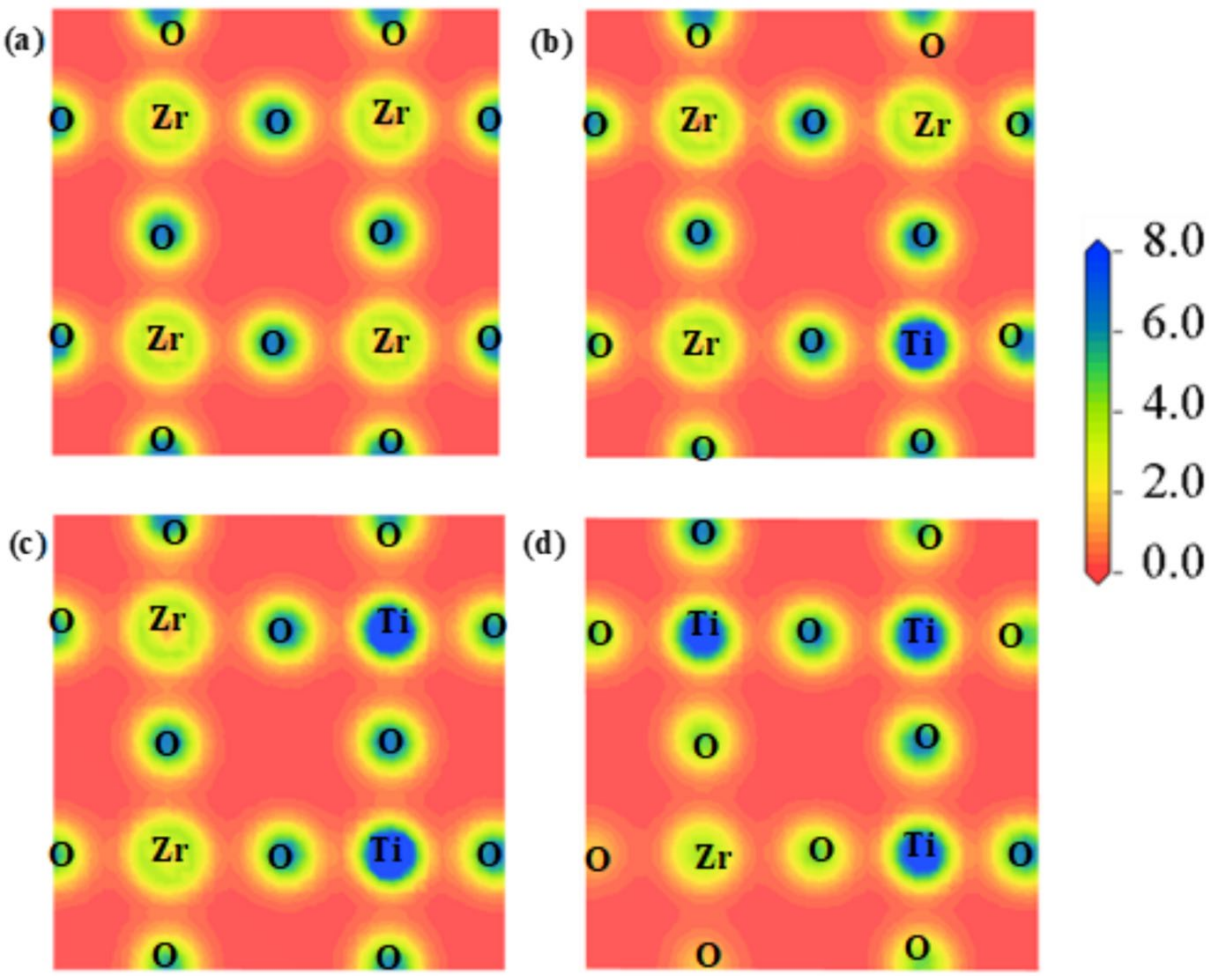
Charge density map of BaZr_1-x_Ti_x_O_3_ along (200) plane where (**a**) x = 0, (**b**) x = 0.25, (**c**) x = 0.5, and (**d**) x = 0.75.

In order to determine the type of chemical bond between atoms, charge density must be calculated. Higher electronegativity atoms have a tendency to draw electron density towards themselves^74^. Figure 4 illustrates the charge density of various compounds along the (2 0 0) plane, elucidating the bonding properties between different atoms in the supercell. The density of electrons is represented by a scale on the right side of the charge density plot. It is visible that the charge density is higher around the O atoms than Ba/Zr in the pure BZO structure as depicted in Fig. 4(a). After Ti doping, the electron cloud increases in Ti and decreases in O atoms. Figure 4 clearly illustrates the covalent nature of the Ti–O/Zr-O bond within all structures. This is characterized by the overlapping and polarization of electron clouds between the bonding atoms, leading to a noticeable increase in covalent bond strengths.

### Optical properties

The energy loss function, absorption coefficient, complex dielectric constant, refractive index, and reflectivity must be calculated to investigate the optical properties of a material. In this study, an in-depth investigation of the optical properties is conducted by comparing pristine BZO with BZO materials doped at various levels. The mathematical equations describing real dielectric function (${\varepsilon }_{1}$), imaginary dielectric function (${\varepsilon }_{2}$), reflectivity $R\left(\omega \right)$, loss function $L\left(\omega \right)$, absorption coefficient $\alpha \left(\omega \right)$, refractive index n(ω), and conductivity ($\sigma$) have been presented below^75–79^. Here, M is the Cauchy principal value operator, V is the normalization factor, ω denotes the angular frequency of the incident light, m is the electron mass, and E_nk_, E_mk_ represent the energy eigenvalues of the bands n and m at a given wavevector k, and ħ is the planck’s constant

1 $${\varepsilon }_{1}=1+\frac{2}{\pi }M\underset{o}{\overset{\infty }{\int }}\frac{{\varepsilon }_{2}\left({\omega }^{1}\right){\omega }{\prime}}{{{\omega }{\prime}}^{2}-{\omega }^{2}} d\omega$$

2 $${\varepsilon }_{2}\left(\omega \right)=\frac{V{e}^{2}}{2\pi \hslash {m}^{2}{\omega }^{2}}\int {d}^{3}k\sum_{n{n}^{\prime}}{\left|\langle kn\left|p\right|kn\rangle ^{\prime}\right|}^{2}f\left(kn\right)\times \left(1-f\left(k{n}^{\prime}\right)\right)\delta \left({E}_{kn}-{E}_{k{n}^{\prime}}-\hslash \omega \right)$$

3 $$\alpha \left(\omega \right)={\left(\sqrt{{\varepsilon }_{1}^{2}\omega +{\varepsilon }_{2}^{2}\omega }-{\varepsilon }_{1}\left(\omega \right)\right)}^\frac{1}{2}$$

4 $$n\left(\omega \right)= \frac{1}{\sqrt{2}}{\left(\sqrt{{\varepsilon }_{1}^{2}\omega +{\varepsilon }_{2}^{2}\omega }-{\varepsilon }_{1}\left(\omega \right)\right)}^\frac{1}{2}$$

5 $$R\left(\omega \right)={\left|\frac{\sqrt{\epsilon \left(\omega \right)}-1}{\sqrt{\epsilon \left(\omega \right)}+1}\right|}^{2}$$

6 $$\sigma =\frac{\alpha nc }{4\pi }$$

7 $$L\left(\omega \right)=\frac{{\varepsilon }_{2}\left(\omega \right)}{\left({\varepsilon }_{1}{\left(\omega \right)}^{2}+{\varepsilon }_{2}{\left(\omega \right)}^{2}\right)}$$

Frequency-dependent optical properties of pure and Ti-doped BZO perovskite have been calculated at energies between 0 and 50 eV. According to the energy spectrum, the visible spectrum shows that light possesses energy ranging from 2.75 eV to 5.2 eV, while the infrared zone of light spans roughly 0–2.75 eV. Energy levels higher than 5.2 eV but lower than 30 eV are accounted for in the ultraviolet (UV) region of light.

**Fig. 5:**
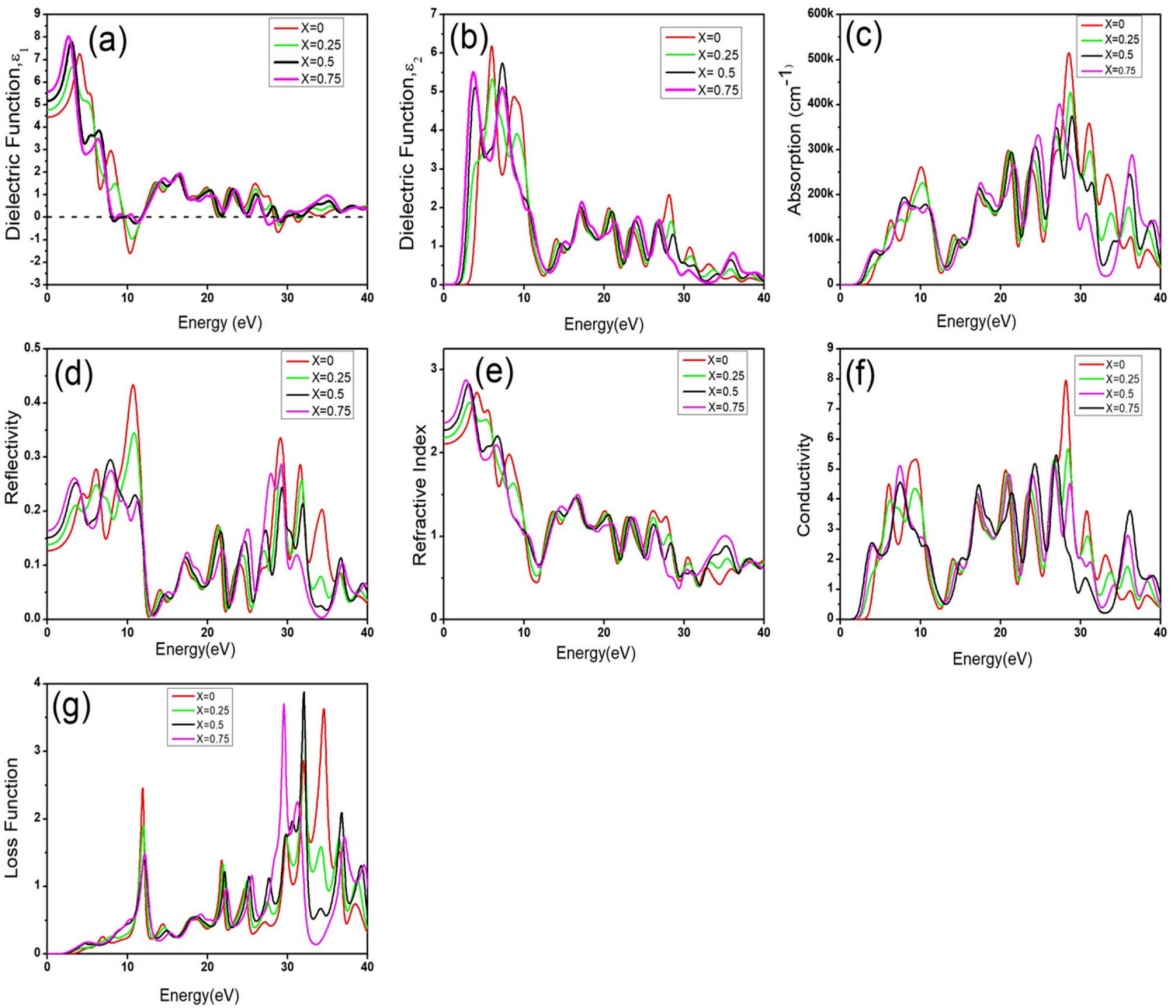
Optical properties of BaZr_1-x_Ti_x_O_3_: (**a**) Real Dielectric Function, (**b**) Imaginary Dielectric Function, (**c**) Absorption coefficient, (**d**) Reflectivity, (**e**) Refractive index, (**f**) Conductivity, and (**g**) Loss function.

The dielectric function plays a pivotal role in assessing how a material interacts with electromagnetic radiation. It is used to characterize physical characteristics by reflecting the information between the energy band structure and optical spectrum lines. A heightened dielectric constant reduces the recombination rate of charge carriers, thereby enhancing the overall efficiency of devices. Consequently, the dielectric function is a crucial material property that significantly impacts how effectively optoelectronic applications operate^80^. The real and imaginary parts of the dielectric functions are depicted in Fig. 5(a) & (b). The complex dielectric function is composed of two fundamental components: the imaginary dielectric function (IDF) ε_2_, which is responsible for energy dissipation, and the real part ε_1_, which represents the ability to store charge. The static dielectric function ε_1_ (0) refers to the real dielectric function ε_1_(ω) evaluated at 0 eV energy. Figure 5(a) illustrates how the static dielectric constant rises as the doping concentration increases. The maximum static dielectric constant has been found at the doping concentration (x = 0.75).

When photon energy increases, the peak value of ε_1_ for pure BZO has been found at 4 eV, while all the doped BZO materials show the highest value below 4 eV. Once they reach their peak, a downward trend has been observed that continues until the negative value of ε_1_(ω). Photon energy ranges of 7–12 eV and 27–30 eV are characterized by negative real dielectric function for both pure and doped BZO materials, implying that the materials do not permit the transmission of electromagnetic waves within these specific energy spectrums of negative ε_1_(ω) values. The metallic behavior of these materials is demonstrated by the actual component of this negative dielectric value. For both pure and doped BZO materials, the IDF is zero at 0 eV, meaning that no energy has been dissipated in these materials at 0 eV. It should be noted that the value of IDF remains zero until absorption begins, which happens when the photon’s energy reaches the band gap energy. All of the pure and doped BZO materials exhibit their first pick of IDF at a photon range between 2–10 eV. These peaks correspond to interband transitions between the VBM and CBM, as this transition governs photon absorption, especially in the context of charge excitations and optoelectronic behavior, as evidenced by the earlier studies^81–83^. Consequently, the probability of an electron transitioning from the valence band to the conduction band is influenced by the magnitude of the imaginary part of the dielectric function. Here, O-2p to Zr-4d, Ti-3d transitions are responsible for causing the IDF peak.

**Fig. 6:**
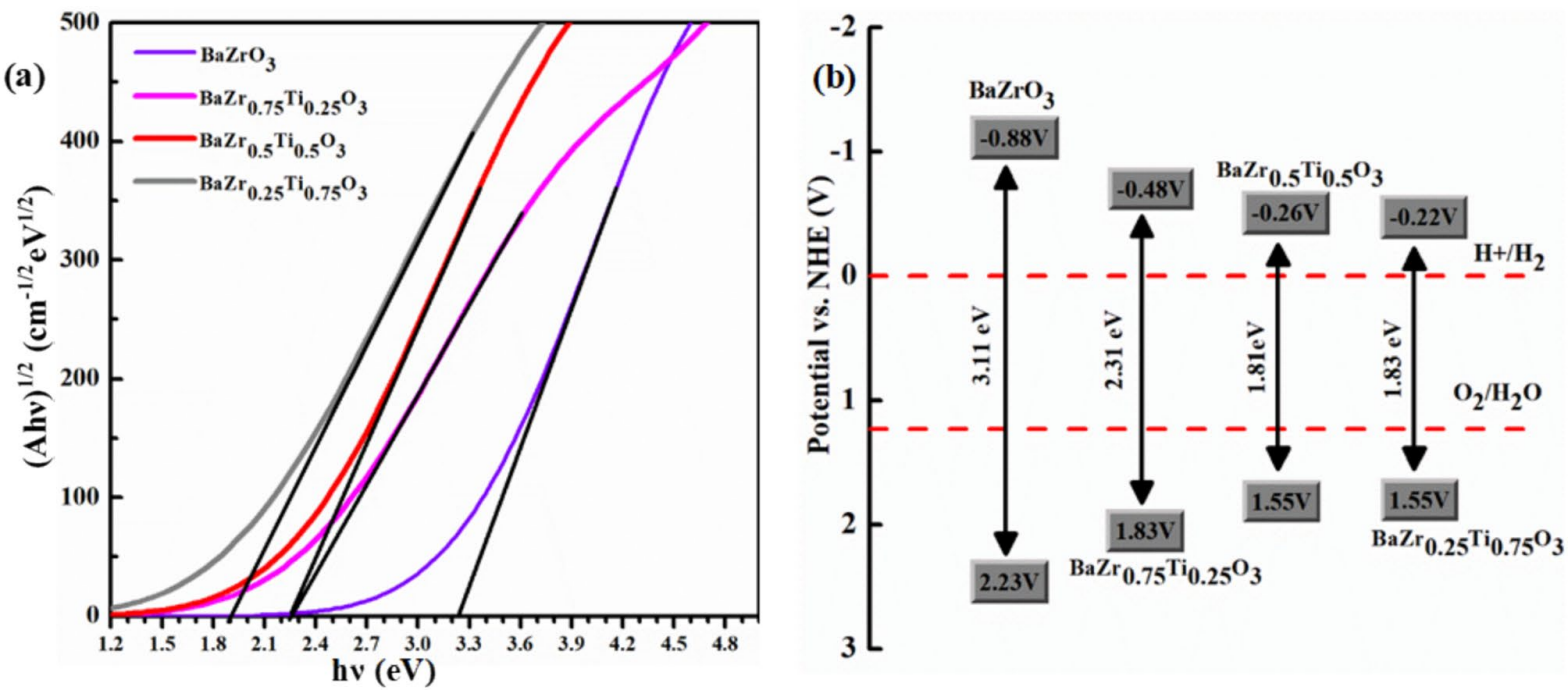
(**a**) Tauc’s plot showing the optical bandgap, and (**b**) the theoretically calculated band edge potentials for BaZr_1-x_Ti_x_O_3_ (x = 0, 0.25, 0.5, 0.75).

**Fig. 7:**
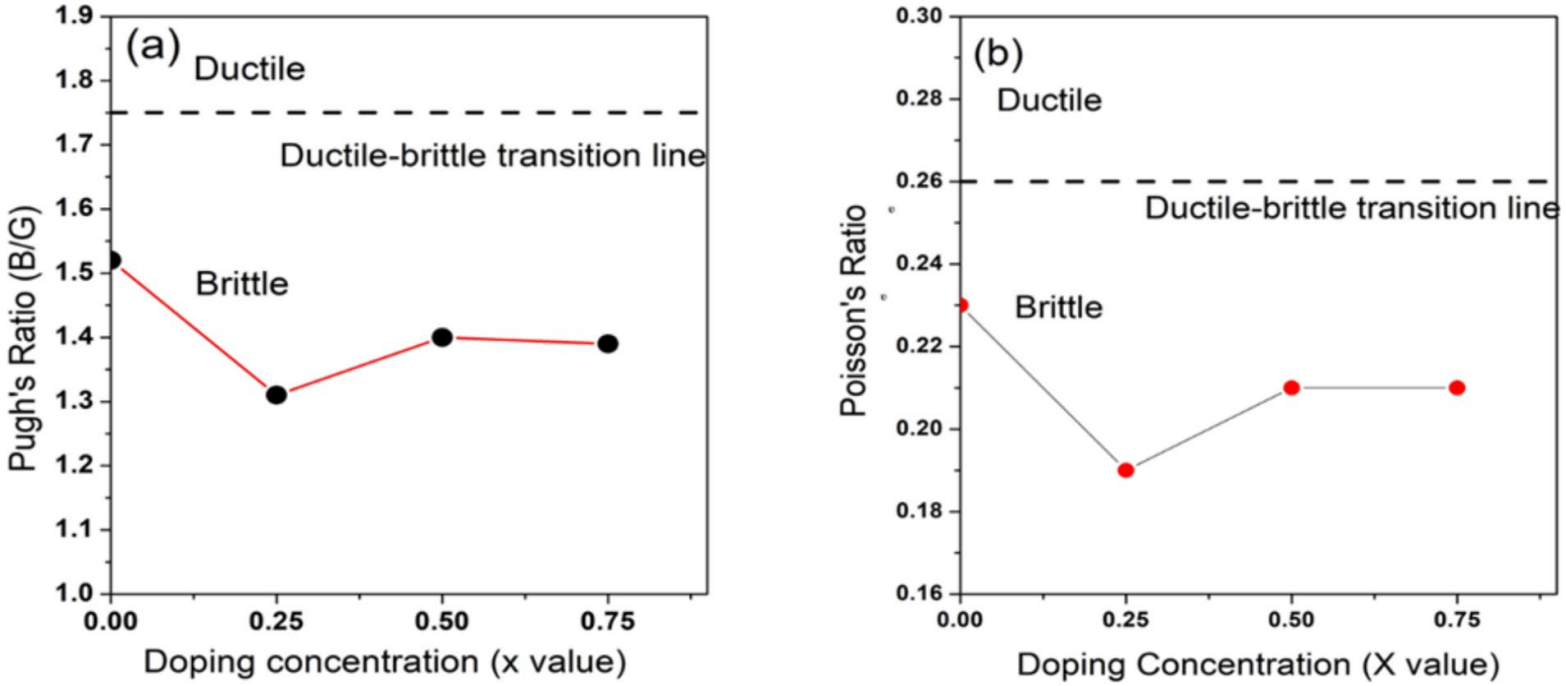
Paugh’s ratio and Poisson’s ratio alternating for BaZr_1-x_Ti_x_O_3_ where (x = 0, 0.25, 0.5, and 0.75).

Absorption has not been observed for both pure and doped BZO at 0 eV. Since bandgap energy is related to absorption, it begins at different photon energies because of the distinct bandgaps of pure and doped BZO materials. The substantial 3.118 eV optical bandgap exhibited by pure BZO restricts its activation solely by UV light. However, reducing this bandgap by Ti doping with different concentrations renders them active not just under UV light but also under visible light. As shown in Fig. 5(c), the doped materials, having smaller bandgaps compared to the pure ones, exhibit absorption onset at lower photon energies, falling within the visible spectrum. Here, the optical bandgap is also determined using a Tauc plot based on the Kubelka–Munk function, as illustrated in Figs. 6(a), 7(a). By extrapolating the linear region of the (αhν)^2^ vs. hν plot to the hν-axis, the intersection point gives the optical bandgap. The estimated optical bandgap values for BaZr_1-x_Ti_x_O_3_ (x = 0, 0.25, 0.5, 0.75) composition are 3.23, 2.25, 2.25, and 1.9 eV, respectively. These values are close to the calculated electronic bandgaps, except for x = 0.5 composition. It is evident that Ti doping at varying concentrations leads to a decrease in the optical bandgap and a corresponding redshift in the absorption peak. The shift in absorption from the UV to the visible range confirms the potential of this material as a photocatalyst for visible-light-driven water splitting and solar cell applications. Bandgap reduction plays a crucial role in enhancing photon absorption, which is essential for solar cell performance as it contributes to increased photocurrent. For water splitting, when a semiconductor absorbs photons, electrons in the valence band (VB) are excited to the conduction band (CB), leaving behind holes in the VB. These electron–hole pairs migrate to the catalyst surface, where water molecules undergo redox reactions to produce H_2_ and O_2_^84^. Since the positions of the conduction and valence band edges relative to the redox potentials of H₂O/O₂ and H⁺/H₂ determine a material’s photocatalytic activity, we have calculated these band edge potentials using the empirical Mulliken electronegativity formula^85^.

8 $${E}_{CBM}={\chi }_{GM}+{E}_{0}-\frac{{E}_{g}}{2}$$

9 $${E}_{VBM}={\chi }_{GM}+{E}_{0}+\frac{{E}_{g}}{2}$$

where ${\chi }_{GM}$ is the geometric mean of the Mulliken electronegativities and E_0_ = 4.5 eV is the NHE potential with respect to the vacuum. ${\chi }_{GM}$ for BaZrO_3_ and BaZr_1-x_Ti_x_O_3_ can be calculated by following relations:

10 $${\chi }_{GM\left({\text{BaZrO}}_{3}\right)}={\left({\chi }_{Ba}.{\chi }_{Zr}.{{\chi }_{O}}^{3}\right)}^\frac{1}{5}$$

11 $${\chi }_{GM\left({\text{BaZr}}_{1-\text{x}}{\text{Ti}}_{\text{x}}{\text{O}}_{3}\right)}={\left({\chi }_{Ba}.{{\chi }_{Zr}}^{1-x}.{{{{\chi }_{Ti}}^{x}.\chi }_{O}}^{3}\right)}^\frac{1}{5}$$

Here, the absolute electronegativity of component atoms Ba, Zr, Ti, and O is denoted by ${\chi }_{Ba}$, ${\chi }_{Ba}$, and ${\chi }_{Ba}$, and ${\chi }_{Ba}$, respectively. To effectively harness visible light for water splitting, the semiconductor should have a band gap below 3.0 eV. Additionally, the CBM must lie above the H^+^/H_2_ reduction potential (0 V), and the VBM must lie below the O_2_/H_2_O oxidation potential (1.23 V) vs. NHE. The calculated results and their relevance are illustrated schematically in Figs. 6(b), 7(b). According to Figs. 6(b), 7(b), the CBM shifts downwards and the VBM shifts upwards, indicating improved photo-reduction and photo-oxidation capacities. Based on the optical bandgap shown in Figs. 6(a), 7(a) and the absorption spectra in Fig. 5(c), the Ti-doped BZO with x = 0.5, x = 0.75 can effectively absorb both visible and UV light, indicating strong potential for solar-driven water splitting and hydrogen production.

The assessment of a material’s surface behavior involves measuring its reflectivity, determined by the ratio of incoming power to the reflected power. Reflectivity is also an important characteristic of materials utilized in solar cell applications. Figure 5(d) illustrates that pure BZO exhibits a zero-frequency limit of 0.126 for reflectivity. From this base value, the reflectivity slightly rises and reaches the maximum value of 0.4. In the case of doped BZO structures, it initiates above the value of 0.126 and attains its maximum value at a photon energy of around 8–10 eV. Notably, owing to collective plasma resonance, the doped materials showcase their lowest reflectivity within 12 eV to 23 eV. Surprisingly, the highest reflectivity coincides with the instance where the ε_1_(ω) dips below zero. This transition into negative values for the ε_1_ signifies a metallic nature in the material. Reflectivity tends to increase with the metallic properties of a compound, hence reaching its peak when ε_1_ exhibits negativity^86^. As shown in Fig. 5(d), the reflectivity is low in the IR–visible region (0–5 eV), indicating that these materials can effectively transmit visible light. This also suggests their potential use in solar cell devices to enhance photocurrent generation and overall efficiency.

The phase velocity of light in a medium is reduced because the material’s electrons collectively polarize and alter its permittivity. This slower phase velocity manifests as a refractive index greater than one. A substance’s refractive index increases with increased electron density. Covalent bonds usually have higher indices because they share electrons, while ionic compounds have lower refractive indices because fewer electrons interact with light photons^87^. The real and imaginary parts of the dielectric constant determine the refractive index, as shown in Eq. 4. In covalent materials, sharing electrons between different atoms leads to more easily polarized molecules under an external electric field. This also leads to a higher value of the real component of the dielectric constant, and thus a higher refractive index^88^. In terms of ionic compounds, there are robust coulomb interactions among ions with opposite charges. Indeed, they become polarized in the presence of an external electric field; however, electrons exhibit greater localization compared to covalent compounds. This leads to a reduction in the real part of the dielectric constant and the refractive index^89,90^. The ε_1_(ω) gives an idea about the refractive index of a material. Refractive index with respect to photon energy ranging from 0 to 50 eV has been depicted in Fig. 5(e). This statement is supported by the charge density map in Fig. 4, which shows that with increasing levels of doping, the covalent character of the bonds increases, leading to rise in the refractive index. At 0 eV photon energy, the refractive index is called static refractive index which is found for pure BZO is 2.10. As doping concentration increases static refractive index of these doped materials also increases.

When intense electromagnetic radiation interacts with a material’s surface, optical conductivity offers information on how bonds are disrupted. Through a band-to-band separation of the IDF, the optical conductivity spectrum can be used to determine the specific transitions responsible for these peaks^91^. The optical conductivity mirrors the trend seen in the absorption coefficient, as illustrated in Fig. 5(f). The peak conductivity has been observed in pure BZO material at photon energy around 28 eV, whereas the doped materials do not show significantly higher conductivity at a specific photon energy. It is noted that at photon energy around 4 eV, all the structures exhibit comparatively lower conductivity. The substantial magnitude of the optical conductivity signifies the material’s exceptional responsiveness to light, indicating a notably heightened photo-response nature in the UV region.

When fast electrons traverse through a material, understanding the loss function is crucial. The zenith of this loss function marks the plasma frequency, denoted as ω*p*, which correlates with the material’s plasma resonance. Plasma frequency (ω_*p*_) is directly linked to the electron density within the material. Notably, photons possessing energies below 2.5 eV do not cause any significant energy loss. Once the photon energy exceeds this threshold, energy loss increases gradually. Both pure and doped BZO materials exhibit their primary peak at photon energy around 11–12 eV as depicted in Fig. 5(g). However, more peaks have been observed in both pure BZO structures at higher photon energies.

### Mechanical properties

The mechanical properties of a material can be analyzed through the utilization of elastic constants, denoted as C_ij_. These constants elucidate how stress induces deformation in a material and how the material reverts back to its original form upon cessation of the stress^92^. Since elastic constants provide the specific mechanical behavior and bonding properties of solids, understanding their independent elastic constants is necessary to comprehend the mechanical behavior of materials. In particular, these elastic constants describe the brittleness, anisotropy, ductility, stiffness, and stability of a material^93^. All calculated elastic constants of different compositions of BZO have been provided in Table 3**,** and the Bulk modulus, Pugh ratio, Poisson ratio, and Anisotropy factor have also been provided in Table 4.

**Table 3:** Independent elastic constants of Zr doped BaZrO_3_.

Composition	C_11_	C_12_	C_13_	C_22_	C_23_	C_33_	C_44_	C_55_	C_66_	C_12_-C_44_
X = 0	264.65	49.26	49.26	264.65	49.26	264.65	65.00	65.00	65.00	−15.74
X = 0.25	227.46	60.00	68.35	227.46	68.35	243.57	98.07	98.07	97.87	−38.07
X = 0.5	258.65	67.97	67.93	258.65	67.93	215.17	93.18	93.18	85.75	−25.21
X = 0.75	236.21	77.76	71.65	236.21	71.65	274.57	101.02	101.02	97.11	−23.26

**Table 4:** Bulk modulus, shear modulus, elastic modulus, Poisson’s ratio, hardness, and anisotropic factor for Ti-doped BaZrO_3_.

Composition	B_V_	B_R_	B	G_V_	G_R_	G	B/G	ʋ	E	H_v_	A
X = 0	121.02	121.01	121.01	81.81	77.11	79.46	1.52	0.23	195.58	14.31	0.61
X = 0.25	121.06	120.79	120.93	92.23	91.62	91.93	1.31	0.19	220.03	19.10	1.17
X = 0.5	120.26	119.15	119.70	85.37	84.81	85.09	1.40	0.21	206.38	16.48	0.98
X = 0.75	132.12	131.72	131.92	94.89	94.06	94.48	1.39	0.21	228.81	18.27	1.27

To ensure mechanical stability, it’s crucial to confirm that each elastic constant adheres to the Born stability criteria and remains positive^94,95^. The cubic structure features three distinct elastic constants, while the tetragonal structure presents six. The computed values of C_11_, C_44,_ and C_44_ for pristine BZO are closely aligned with previously calculated values^96^. The Born stability criteria govern the material’s mechanical behavior and are summarized by the following relationships^93,97–101^:

*C*_11_ > 0, *C*_11_ + 2*C*_12_ > 0, *C*_44_ > 0, *C*_11_ − *C*_12_ > 0 (Stability criteria for cubic structure).

*C*_11_ > $\left|{C}_{12}\right|$, C_66_ > 0, C_11_ + C_33_ − 2C_13_ > 0, C_11_ − C_12_ > 0, 2(C_11_ + C_12_) + C_33_ + 4C_13_ > 0) (Stability criteria for Tetragonal structure).

According to this investigation, every structure met the above-mentioned conditions, confirming the materials’ stability at various doping levels. The tetragonal structure (X = 0.5) exhibits a slightly lower C_11_ value compared to C_33_, indicating weaker compressibility along the [001] direction than the [100] direction. Conversely, in the structure (X = 0.25,0.75), the C_11_ value is slightly lower than C_33_, suggesting higher chemical bonding energy in the [010] and [100] directions relative to the [001] direction. Across all tetragonal structures, C_44_ surpasses C_66_, highlighting the tendency for easier shear deformation along the [010] direction than the [001] direction^102,103^. Key mechanical properties, such as elastic moduli (B, G, and E), Poisson’s ratio (v), Pugh’s ratio, and anisotropy, have been calculated using the Reuss and Voigt techniques with elastic constants. Additionally, the hardness (Hv) and factor (A) of pristine and doped BZO are examined in this study. For cubic structures, the Voigt-Reuss-Hill scheme provides an approach for approximating bulk and shear moduli. Utilizing the Vigot and Resus theory, the bulk modulus is determined by employing Eq. 8 for cubic structures, while Eqs.13 & 14 are employed for tetragonal structures^92,104,105^. Here, C_ij_ refers to the elastic constants and S_ij_ refers to the elastic compliance constants.

12 $$B={B}_{v}={B}_{R}=\frac{{C}_{11}+2{C}_{12}}{3}$$

13 $${B}_{R}=\frac{1}{\left({s}_{11}+{s}_{22}+{s}_{33}\right)+2\left({s}_{12}+{s}_{13}+{s}_{23}\right)}$$

14 $${B}_{v}=\frac{{C}_{11}+{C}_{22}+{C}_{33}}{9}+\frac{2\left({C}_{12}+{C}_{13}+{C}_{23}\right)}{9}$$

According to the Vigout and Resus theory, the shear modulus is computed using Eqs.15 & 16 for cubic structures, and Eqs.17 & 18 for tetragonal structures^106^.

15 $${G}_{v}=\frac{{C}_{11}-{C}_{12}+3{C}_{44}}{5}$$

16 $${G}_{R}=\frac{5{C}_{44}\left({C}_{11}-{C}_{12}\right)}{4{C}_{44}+3\left({C}_{11}-{C}_{12}\right)}$$

17 $${G}_{v}=\frac{{(C}_{11}+{C}_{22}+{C}_{33}-{C}_{12}-{C}_{13}-{C}_{23} )}{15}+\frac{\left({C}_{44}+{C}_{55}+{C}_{66}\right)}{5}$$

18 $${G}_{R}=\frac{1}{4\left({s}_{11}+{s}_{22}+{s}_{33}\right)-4\left({s}_{12}+{s}_{13}+{s}_{23}\right)+3({s}_{44}+{s}_{55}+{s}_{66})}$$

For cubic pristine BZO, the anisotropic factor (A) is determined using Eq.19, while Eq.20 is utilized for tetragonal structures^107,108^.

19 $$A=\frac{2{C}_{44}}{{C}_{11}-{C}_{12}}$$

20 $$A=\frac{4{C}_{44}}{{C}_{11}+{C}_{33}-{C}_{13}}$$

Several general equations (Eqs.21–25) exist for calculating Young’s modulus ($E$), Hill’s shear modulus ($G$), Hill’s bulk modulus ($B$), Poisson’s ratio ($\nu$), and Hardness (${H}_{\nu }$) for both cubic and tetragonal structures based on the aforementioned Equations^109,110^.

21 $$G=\frac{{G}_{v}+{G}_{R}}{2}$$

22 $$B=\frac{{B}_{v}+{B}_{R}}{2}$$

23 $$E=\frac{9BG}{3B+G}$$

24 $$\nu =\frac{3B-2G}{2\left(3B+G\right)}$$

25 $${H}_{\nu }=\frac{\left(1-2\nu \right)E}{6\left(1+\nu \right)}$$

The ductility testing proposed by B/G is called the Paugh ratio, with a threshold value set at 1.75. To determine a material’s ductility or brittleness, Pugh’s ratio is used. When a material’s B/G ratio is greater than 1.75, it is classified as ductile; if not, it is called as brittle^111^. As seen in Figs. 6(a), 7(a), all the compounds investigated in this work had a Paugh’s ratio of less than 1.75, showing that they are inherently brittle.

**Fig. 8:**
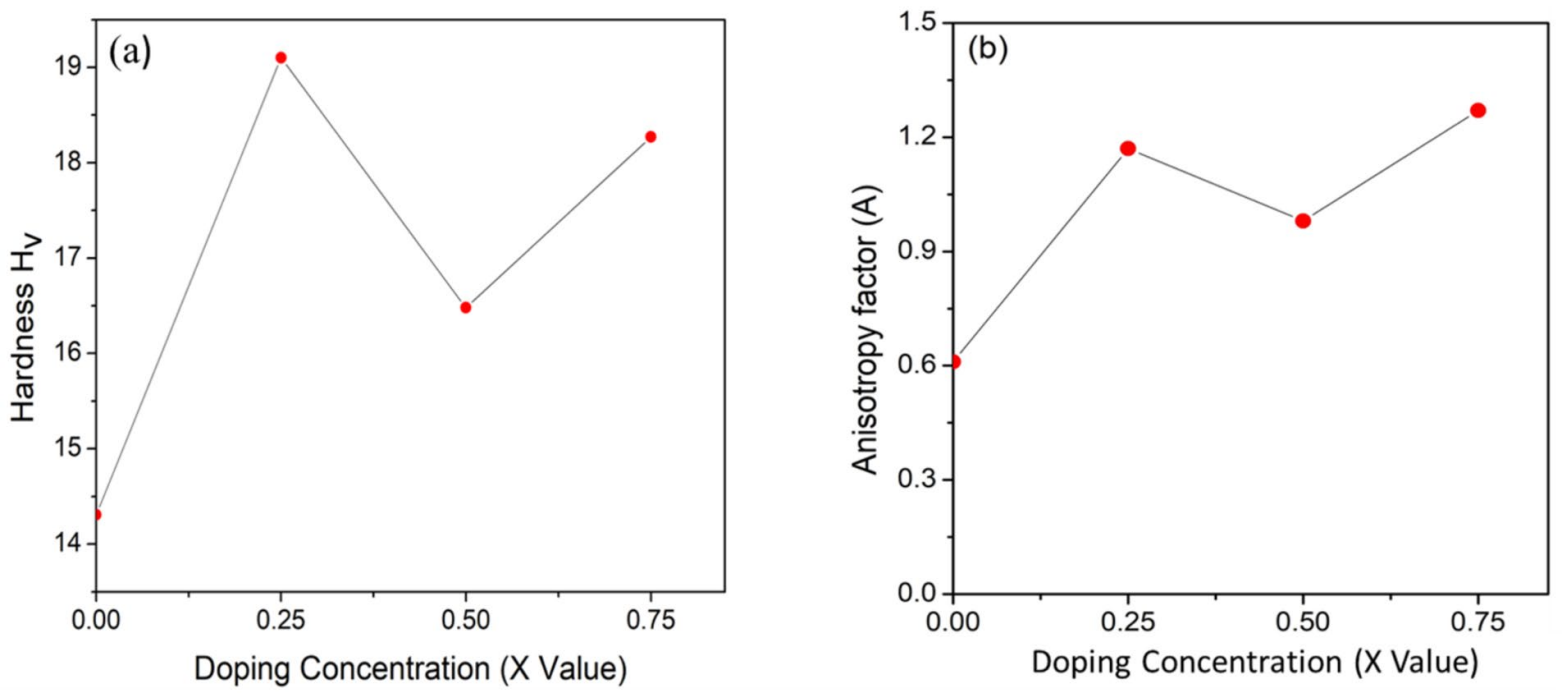
Paugh’s ratio and Poisson’s ratio alternating for BaZr_1-x_Ti_x_O_3_ where (x = 0, 0.25, 0.5, and 0.75).

Poisson’s ratio ($\nu$) is also a crucial parameter for characterizing the mechanical behavior of materials. It serves as a key indicator for assessing ductility versus brittleness. Additionally, Poisson’s ratio ($\nu$) is a parameter in evaluating the shear stability of a lattice structure; higher values typically signify superior plasticity^112^. Materials with a Poisson’s ratio greater than 0.26 are generally classified as ductile, while those with values below this threshold are considered brittle^113,114^. Materials with a Poisson’s ratio greater than 0.26 are generally classified as ductile, while those with values below this threshold are considered brittle^113,114^. The Poisson’s ratio consistently stayed below the ductile–brittle transition line at various doping levels, indicating the brittleness of these structures as depicted in Fig. 6(b), 7(b). The hardness of a polycrystalline material refers to its ability to withstand external forces, making it a vital characteristic to explore, especially for materials employed in demanding industrial applications. fluctuation in hardness with respect to doping concentration is shown in Fig. 8(a), demonstrating the maximum hardness at a doping concentration of (x = 0.25).

The value of A is fixed to 1 for an isotropic compound; on the other hand, it becomes larger or smaller for an anisotropic compound. The degree of elastic anisotropy in crystals is indicated by the deviation of this value from 1^115^. Table 3 illustrates that there is no unity value across all the structures, indicating all the structures (x = 0, 0.25, 0.5, 0.75) are anisotropic. Figure 8(b) distinctly illustrates the variation of the anisotropy factor concerning different doping concentrations.

### Thermodynamic properties

**Fig. 9:**
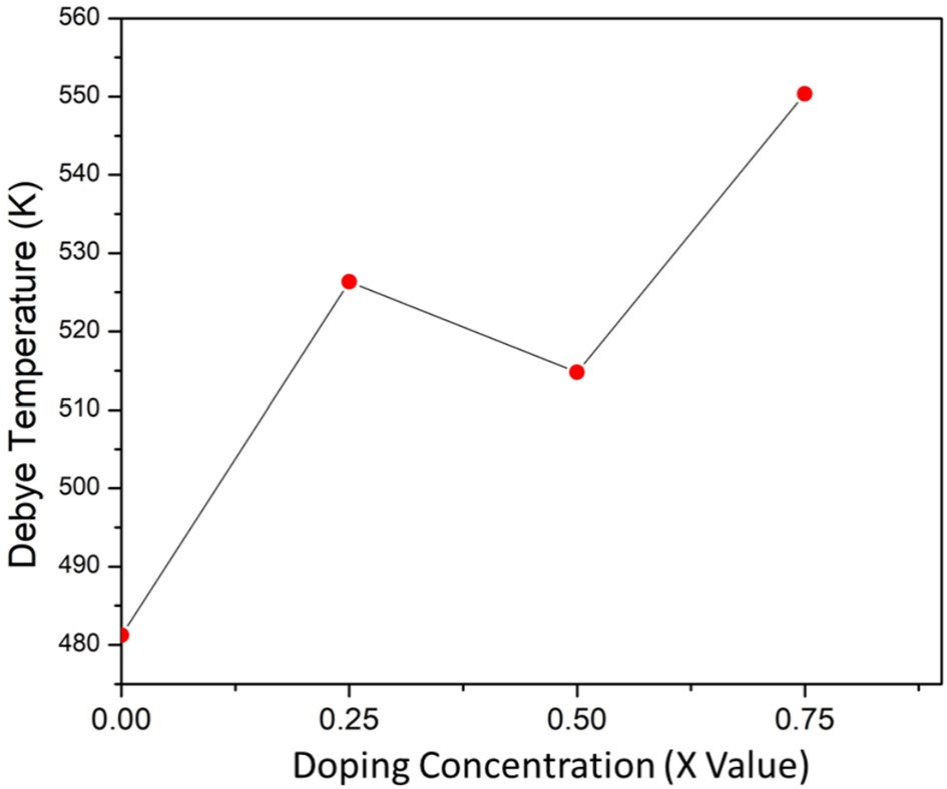
Variation of Debye Temperature in BaZr_1-x_Ti_x_O_3_ where (x = 0, 0.25, 0.5, and 0.75).

Debye temperature is a crucial parameter that characterizes the strength of atomic bonds within crystals, wherein the maximum frequency of thermal vibrations in a solid is directly linked to its Debye temperature. The following is the established relationship between average wave velocity and Debye temperature^116,117^:

26 $${\theta }_{D}=\frac{h}{k}{\left(\frac{3n{N}_{A}\rho }{4\pi M}\right)}^{\raisebox{1ex}{$1$}\!\left/ \!\raisebox{-1ex}{$3$}\right.}{v}_{m}$$

where the molecular weight ($M$), density ($\rho$), Avogadro constant (${N}_{A}$), Planck constant ($h$), and Boltzmann constant ($k$). Debye temperature has a substantial influence on thermal conductivity, lattice vibration, melting temperature, specific heat, and thermal expansion. The change in Debye temperature with different doping concentrations has been depicted in Fig. 9. The maximum Debye temperature has been observed at a higher doping concentration of (x = 0.75). As the doping level increases, a higher Debye temperature suggests increased strength in covalent bonds, resulting in decreased pliability^118^. The ${\theta }_{D}$ values of the various structures (x = 0, 0.25, 0.5, 0.75) are close to the well-known thermal barrier coating (TBC) material Y_4_Al_2_O_9_ (${\theta }_{D}=564 K$)^119^. This implies that the previously mentioned structures with doping concentration (x = 0, 0.25, 0.5, 0.75) would be a better choice for TBC materials.

## Conclusion

This study investigates the physical properties of pure BZO and Ti-doped BZO at different doping contents, where DFT has been as been used with the exchange–correlation function of GGA and PBE. Electronic band structures of pure and doped BZO both show indirect energy gaps. In pure BZO, the uppermost portion of the VB arises from the hybridization of O-p states and Ba-p states, while the CB mostly originates from the s-state of Ba and the d-state of Zr. The O-p state dominates the VB of doped BZO, with tiny contributions from the Zr-d and Ti-d states. In contrast, the Ti-d state leads the CB in doped BZO compounds, with a minor contribution from the Zr d states. Various optical properties such as the dielectric function (ɛ_1_ and ɛ_2_), absorption coefficient, conductivity, reflectivity, reflectivity, and loss function have been illustrated in this study. The results revealed distinct changes in the optical properties following the Ti doping in BZO. Remarkably, there is a notable shift observed in the absorption edge towards lower energy regions, indicative of a reduced bandgap in the doped BZO materials compared to the pure BZO. The brittleness of the investigated structures is confirmed by the analysis of Pugh’s ratio and Poisson’s ratio. In addition, the compound’s hardness and machinability index have been determined through extensive analysis to determine its suitability for industrial use. The study suggests that the solids could be used as TBC materials due to their promising mechanical properties. Moreover, with the growing energy crisis driven by the excessive use of fossil fuels, this study holds significant potential for advancing the industrial production of hydrogen. Beyond exploring the promising properties of the investigated materials from a computational and theoretical perspective, this work paves the way for broader experimental research with doped BZO as a promising solar cell absorber and photocatalyst. Further experimental studies are essential to evaluate the suitability of these materials for practical applications such as water splitting and TBC.

## Data Availability

The datasets generated and/or analysed during the current study are not publicly available as the data also forms part of an ongoing study but are available from the corresponding author upon reasonable request.
